# 0589. Pravastatin exerts opposite effects on splanchnic microcirculatory oxygenation during sham or septic conditions in an animal model of polymicrobial sepsis

**DOI:** 10.1186/2197-425X-2-S1-P34

**Published:** 2014-09-26

**Authors:** C Beck, F Barthel, A Herminghaus, C Vollmer, I Bauer, O Picker

**Affiliations:** University Hospital Duesseldorf, Department of Anaesthesiology, Duesseldorf, Germany

## Introduction

In addition to lipid-lowering effects HMG-CoA reductase inhibitors like pravastatin also modulate the microcirculation [1]. The exact mechanisms are yet unknown and results are heterogeneous, with both positive and negative effects on endothelial microvascular function [2, 3] being reported.

## Objectives

The aim of this study was to evaluate the effects of pravastatin on the microcirculatory oxygenation of the colon in a rodent model of polymicrobial sepsis.

## Methods

The data derive from a total of 40 experiments on rats studied with approval of the local animal care and use committee. Pravastatin (0.2 mg/kg) or NaCl were injected subcutaneously 18 h prior to sepsis induction (colon ascendens stent peritonitis) or sham operation. 24 h after induction of sepsis the animals were re-laparotomized under general anaesthesia and received ongoing fluid replacement and pressure-limited ventilation for 120 min. Macrohemodynamic variables were recorded and microcirculatory oxygen supply (µDO_2_) and post-capillary oxygen saturation (µHbO_2_) of the colon were measured simultaneously via laser Doppler and tissue reflectance spectrophotometry, respectively. Data are presented as means±SD, 2-way ANOVA followed by Dunnett (vs. baseline) or Tukey (between groups).

## Results

**Figure 1:**
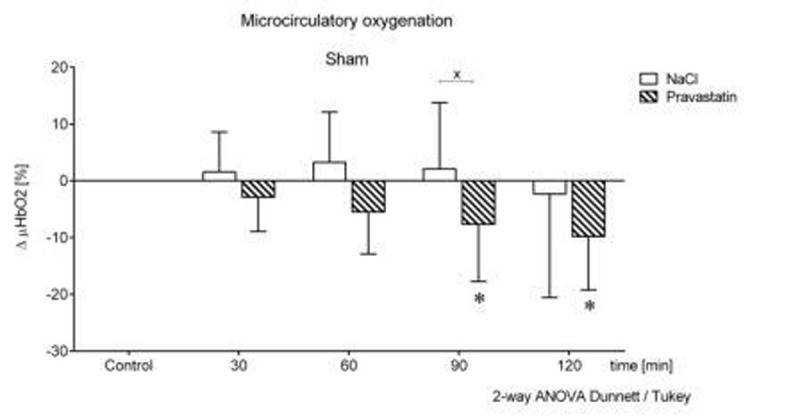
Sham

**1.)** In pravastatin pre-treated sham animals the microcirculatory oxygenation µHbO_2_ declined by 9.8±9.4% with no change in the NaCl group. Figure 1.

**Figure 2:**
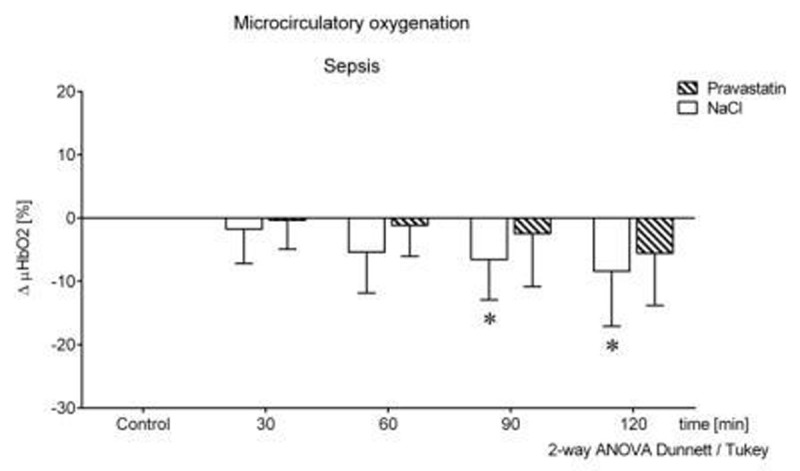
Sepsis

**2.)** During sepsis pravastatin pre-treatment ameliorated the deterioration of µHbO_2_ (-5.5±8.2%), compared to a significant decrease in the NaCl group (-8.4±8.7%). Figure 2.

**3.)** Macrohaemodynamic variables and microcirculatory oxygen supply of the colon did not differ between the groups.

## Conclusion

Pravastatin has opposite effects on splanchnic microcirculatory oxygenation depending on septic or non-septic conditions. These effects are independent of the macrocirculation or microcirculatory oxygen supply.
